# Increased frequency of CD4+ CD25+ FOXP3+ cells correlates with the progression of 4-nitroquinoline1-oxide-induced rat tongue carcinogenesis

**DOI:** 10.1007/s00784-013-1146-5

**Published:** 2013-11-22

**Authors:** Jianjiang Zhao, Zhiping Wang, Jiusong Han, Xiaoling Qiu, Jie Pan, Jun Chen

**Affiliations:** Department of Surgery, Guangdong Provincial Stomatological Hospital, Southern Medical University, Guangzhou, 510280 China

**Keywords:** Treg, Oral squamous cell carcinoma, 4-nitroquinoline 1-oxide

## Abstract

**Objectives:**

CD4+ CD25+ FoxP3+ T cells (Tregs) play an essential role in sustaining self-tolerance by negatively regulating immune responses. Increased frequencies of Tregs have been reported in a variety of human cancers. The aim of this study was to evaluate the prevalence of Tregs infiltration in the peripheral blood and regional lymph nodes during rat tongue carcinogenesis induced by 4-nitroquinoline-1-oxide (4NQO).

**Materials and methods:**

Forty-eight Sprague–Dawley rats were divided into the control (*n* = 16) and experimental groups (*n* = 32) to which 4NQO in drinking water was administered. Flow cytometry was used to analyze the prevalence of Tregs in lymphocytes of peripheral blood and regional lymph nodes during 4NQO-induced rat tongue carcinogenesis. CD4+ CD25+ FoxP3+ cells were expressed as a percentage of the total CD4+ cells.

**Results:**

The frequency of Tregs in peripheral blood from squamous cell carcinoma rats was significantly higher than controls (3.82 ± 0.62 versus 1.40 ± 0.31 %, *P* < 0.001). The proportion of Tregs was sequentially increased from moderate dysplasia to severe dysplasia and SCC (1.94 ± 0.72, 2.29 ± 0.82, and 3.82 ± 0.62 %, respectively). The frequency of Tregs in regional lymph nodes from squamous cell carcinoma rats was also significantly higher than normal rat mucosa (14.67 ± 3.09 versus 5.53 ± 2.07 %, *P* < 0.001). The percentage of Tregs was gradually increased in moderate dysplasia, severe dysplasia, and SCC groups (8.93 ± 1.74, 10.15 ± 0.86, 14.67 ± 3.09 %, respectively) as compared to control group (5.53 ± 2.07 %).

**Conclusion and clinical relevance:**

Tregs in peripheral blood and lymph nodes were associated with disease progression during 4NQO-induced rat tongue carcinogenesis. This study indicated that the upregulation of Tregs might play important role during oral mucosa malignant transformation.

## Introduction

Oral squamous cell carcinoma (OSCC) is the most common oral malignancy with a high propensity to local recurrence, most commonly in the regional lymph node. Despite recent advances in therapy, the 5-year survival rate of OSCC has remained at approximately 50 % [1]. The development of oral cancer is usually preceded by the appearance of premalignant lesions which have the potential to develop into cancer [2]. The mechanisms involved in the transformation of premalignant/ potentially malignant lesions to invasive cancer remain largely unknown. Therefore, improving our knowledge about the precise molecular mechanisms involved in oral mucosa tumorigenesis from premalignant lesions to invasive squamous cell carcinoma is critical to develop new treatment strategies for OSCC.

It is increasingly realized that the host immune system also plays a pivotal role in oral carcinogenesis apart from various factors including tobacco, alcohol, viral infection, and physical irritation [3]. CD4+ CD25+ Foxp3+ regulatory T cells (Tregs) are a unique subset of CD4+ T cells characterized by specific expression of CD25 (the α-chain of the receptor for interleukin-2) and the transcription factor forkhead box protein P3 (Foxp3) [4]. There is accumulating evidence that Tregs play an important role in the suppression of antitumor immune responses [5, 6]. Several recent studies have reported that increase population of Tregs in peripheral blood, draining lymph nodes, and tumor microenvironment in various cancer patients, including gastric, lung, breast, colorectal, and head and neck squamous cell carcinoma [7–10]. Furthermore, the increase of Tregs is likely to correlate with poor outcome and reduced survival in these cancer patients [11–13]. Gasparoto et al. [14] reported that Tregs were increased in blood and primary tumors of OSCC patients, and these cells appeared to suppress immune responses. These studies demonstrated that Tregs exerted immune suppressive function in tumor progression. However, until now, no report focuses on the distribution of Tregs during multi-step process of oral carcinogenesis.

To explore the role of Tregs and their correlation with multistage carcinogenesis, a good experimental model of 4-nitroquinoline 1-oxide (4NQO)-induced tongue carcinogenesis was used. The rat tongue carcinoma induced by water-soluble 4NQO closely mimics all carcinogenesis stages of their human counterparts from various degrees of epithelial dysplasia to carcinoma, which provides an ideal model for understanding how neoplasm develops [15].

In the current study, we investigated the distribution of CD4+ CD25+ Foxp3+ Tregs in the peripheral blood and regional lymph node lymphocytes during 4NQO-induced rat tongue carcinogenesis and determined their relationships with tumor progression.

## Materials and methods

### Reagents

4NQO obtained from Sigma-Aldrich (St. Louis, MO, USA) was dissolved in distilled water, as stock solution (500 ppm), and diluted in the drinking water to a final concentration of 25 ppm.

### Experimental model

All animal experiments were carried out according to the guidelines of the Committee on Experimental Animals of Sun Yat-sen University (SYU), PR of China. We purchased forty-eight Sprague–Dawley (SD) rats (6 weeks old; 90–95 g) from the Animal Experimental Center of SYU. The animals were maintained under specific pathogen-free conditions of temperature (24 ± 2 °C), light–dark periods of 12 h. The animals were randomly assigned to three groups (*n* = 16 rats per group). In the experimental groups, rats were daily fed with distilled water containing 25 ppm 4NQO for 16 or 24 weeks, whereas animals in the control group were fed with distilled water only. At the end of the experimental period, the rats were anesthetized using isoflurane, and blood was collected by cardiac puncture using BD vacutainer vials containing EDTA. All neck lymph nodes of a reasonable size were harvested in each group. Blood and lymph node samples were sent to a central FACS laboratory and were analyzed for the presence of regulatory T cells. The tongue tissues were collected and longitudinally separated bisected for histopathological examination. The tissue was fixed in 10 % buffered formalin and stained with hematoxylin and eosin (H.E., Merck, Darmstadt, Germany).

### Histopathological examination

For each experimental rat, both the visible lesion and clinically normal adjacent mucosa on the tongue were collected. Histopathological evaluation was performed by two experienced oral pathologists in a double-blind manner. Analyses of the tongue specimens were graded as normal epithelium, mild dysplasia, moderate dysplasia, severe dysplasia, and carcinoma, basically according to the criteria described by Kramer et al. [16].

### Isolation of peripheral blood mononuclear cells

Rat peripheral blood was collected in heparinized tubes and diluted with equal fresh sterile phosphate-buffered saline (PBS). Peripheral blood mononuclear cells (PBMCs) were isolated using Ficoll-Hypaque (eBioscience, San Diego, USA) density gradient centrifugation, washed, and counted. Viability of PBMCs was determined by a trypan blue dye and immediately used for experiments.

### Preparation of lymph node lymphocytes

Lymph nodes were harvested from 4NQO-induced rats and control rats. A single-cell suspension was prepared from the lymph node within 1 h after excision. Tissues were first washed with PBS and cut into 3–5 mm pieces and then disaggregated mechanically into single-cells by a Medimachine (DAKO, Hamburg, Germany). Then, the lymph node suspensions were lysed with FACS^TM^ Lysing Solution (BD Biosciences) for 10 min at room temperature in the dark. After erythrocyte lysis, cells were washed with PBS and counted in a trypan blue dye. The cell concentration was adjusted to 2 × 10^7^/mL.

### Flow cytometry analysis

The following antibodies were used for flow cytometry: anti-CD4-FITC and anti-CD4-PE; anti-Foxp3-APC and their respective isotypes were purchased from eBioscience (San Diego, USA). The PBMCs or lymph node lymphocytes were incubated with a cocktail of FITC-labeled anti-rat CD4 monoclonal antibody and PE-labeled anti-rat CD25 monoclonal antibody for 30 min in the dark at 4 °C to stain the surface. Next, cells were stained intracellularly for Foxp3 according to the manufacturer's instructions. Briefly, cells were resuspended in 1 ml Foxp3 fixation/permeabilization buffer (Fix/Perm, eBioscience) and incubated for 30 min at 4 °C in the dark. Then, Foxp3 antibody or isotype control was added followed by incubation for 30 min at 4 °C in darkness. Finally, cells were washed, resuspended in washing buffer, and analyzed by flow cytometry. Data were acquired on an FC500 flow cytometer (Beckman Coulter) and analyzed with CXP software. A gate was first set on lymphocytes followed by gating of CD4+ T cells. Within this gate, Tregs were defined as CD4+ CD25+ Foxp3+ cells. The frequency of Foxp3+ Treg cells was shown as percent of the total CD4+ lymphocytes.

### Statistical analysis

Statistical analysis was performed by using the SPSS statistical software (SPSS16.0, Chicago, USA). Data are expressed as mean ± SEM. Quantitative densitometry values were compared by Student's *t* test. For multiple group comparisons, one-way ANOVA with Tukey's post test was performed. *P* < 0.05 was considered significant.

## Results

### Histopathological evaluation following 4NQO treatment

**Fig. 1:**
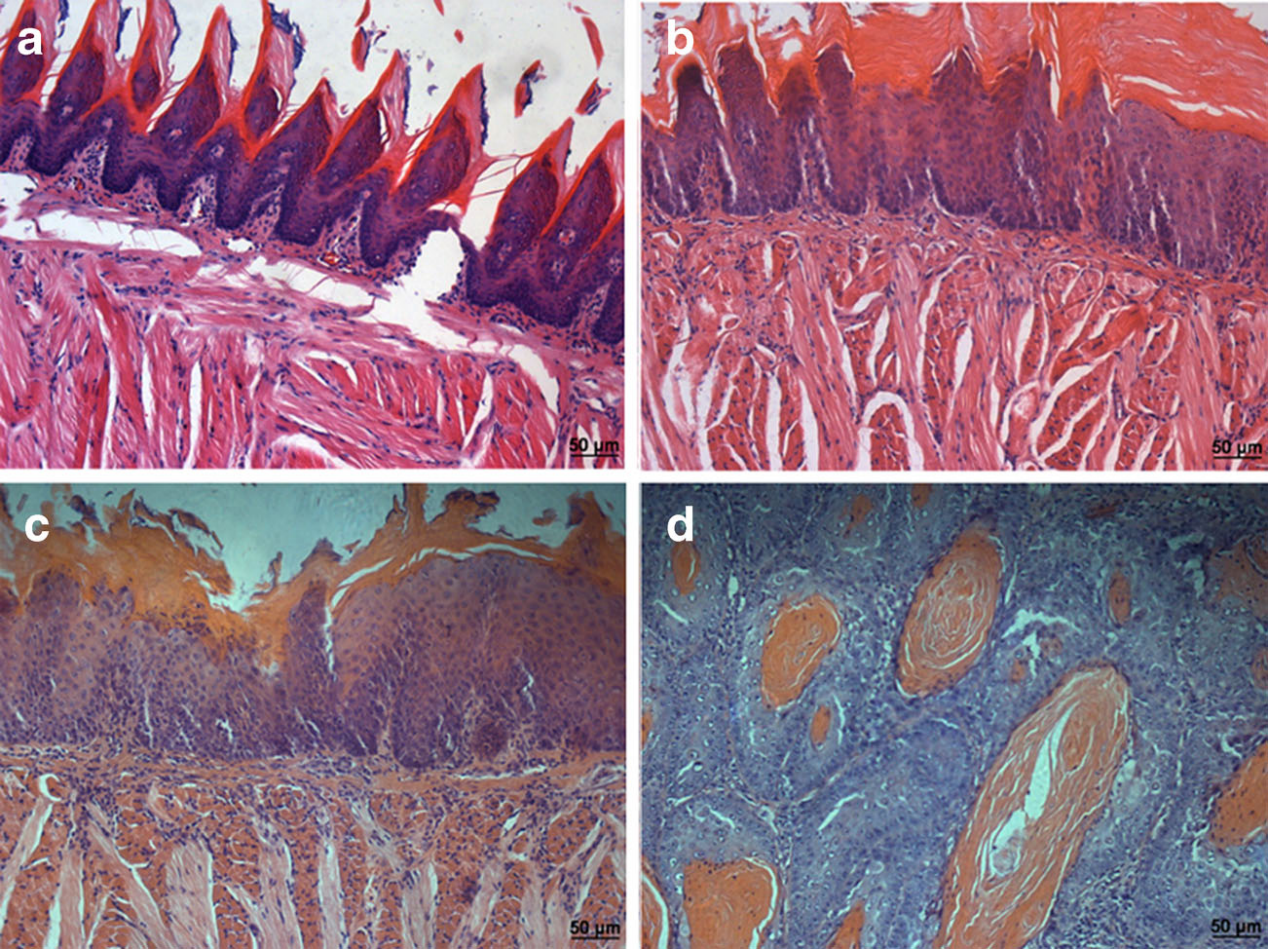
Histopathology of 4-nitroquinoline 1-oxide-induced rat tongue carcinogenesis stained by hematoxylin and eosin. **a** Normal, **b** moderate dysplasia, **c** severe dysplasia, **d** squamous cell carcinoma. Original magnification, ×200. *Bar*: 50 μM

None of the control animals developed any visible tongue epithelia lesions and histopathological changes through the end of the study (Fig. 1a). At 16 weeks, moderate and/or severe dysplasia was found in the tongue (Fig. 1b, c). At 20 weeks, severe oral dysplasia and squamous cell carcinoma (SCC) were found (Fig. 1d). The histopathological grade was usually squamous cell carcinoma of a well-differentiated type. The tumors spread into the submucosa and underlying muscle layer, forming small nests with typical keratin pearl formation. The histopathological findings are summarized in Table 1. According to histological study, specimens were divided into four groups: normal (*n* = 16), moderate dysplasia (*n* = 8), severe dysplasia (*n* = 12), and SCC (*n* = 12).

**Table 1:** Incidence of histopathological lesions in tongue of rats in the 4-nitroquinoline 1-oxide model

Groups (weeks)	No. of rats	Lesions			
		Normal	Moderate dysplasia	Severe dysplasia	Carcinoma
0	16	16	0	0	0
16	16	0	8	8	0
24	16	0	0	4	12

### Distribution of Tregs in rat peripheral blood

**Fig. 2:**
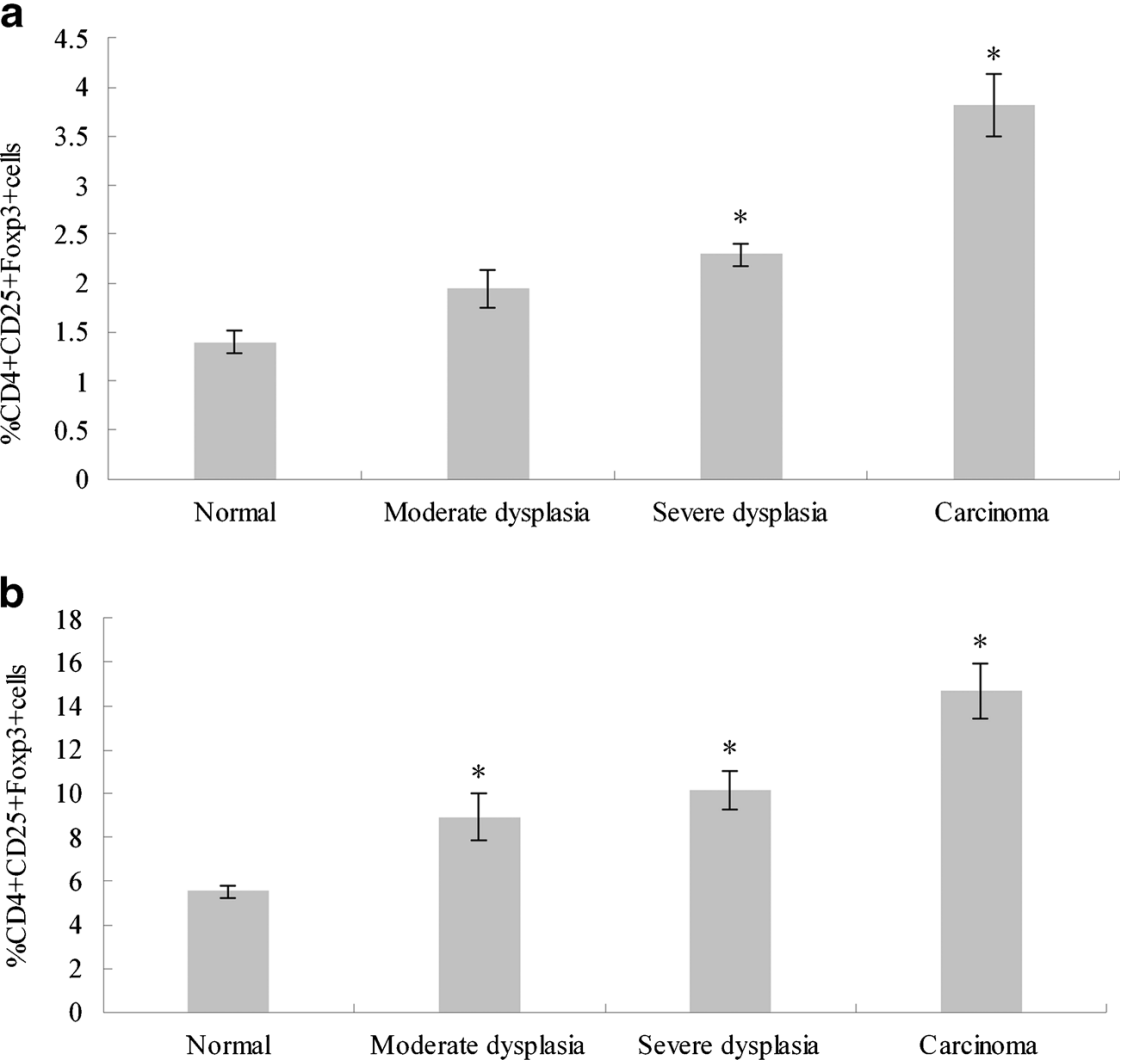
The frequency of CD4+ CD25+ Foxp3+ T cells in peripheral blood and lymph nodes in rats given 4NQO in drinking water and control rats without 4NQO. **a** The prevalence of CD4+ CD25+ Foxp3+ T cells in peripheral blood was gradually increased in 4NQO-induced rats as compared with normal controls. **b** The prevalence of CD4+ CD25+ Foxp3+ T cells in lymph nodes was higher in 4NQO-induced rats than normal controls. The data are reported as mean ± SD of three independent experiments.*P < 0.001 as compared with control

The frequency of Tregs in 4NQO-treated rat peripheral blood as well as in the control group was evaluated by flow cytometry (Fig. 2a). Tregs were identified as CD4+ CD25+ Foxp3+ cells and expressed as a percentage of the total CD4+ cells. The CD4+ CD25+ Foxp3+ cells stepped up along with the progression of 4NQO-induced rat tongue carcinogenesis. The frequency of Tregs in OSCC rat was significantly higher than control (3.82 ± 0.62 versus 1.40 ± 0.31 %, *P* < 0.001). The proportion of Tregs was sequentially increased from moderate dysplasia to severe dysplasia and SCC (1.94 ± 0.72, 2.29 ± 0.82, 3.82 ± 0.62 %, respectively). Statistical differences (*P* < 0.001) were observed between severe dysplasia and SCC groups. These results suggested that Tregs were related to rat tongue squamous carcinoma progression.

### Distribution of Tregs in rat lymph node

The density of Foxp3+ Tregs in rat lymph nodes was higher than that in peripheral blood. The percent of Tregs in rat lymph nodes increased upon the development of tongue carcinogenesis. The frequency of Tregs was gradually increased in moderate dysplasia, severe dysplasia, and SCC groups (8.93 ± 1.74, 10.15 ± 0.86, 14.67 ± 3.09, respectively) as compared to the control group (5.53 ± 2.07 %) (Fig. 2b). The percentage of Tregs was significantly higher in severe dysplasia and SCC rat than in control rat (*P* < 0.001, *P* < 0.001, respectively). SCC rats represented higher proportion of Tregs than those with severe dysplasia (*P* = 0.002). No significant statistically difference (*P* > 0.05) was observed between the severe dysplasia group and moderate dysplasia group (*P* = 0.0279).

## Discussion

In the present study, we have described the sequential analysis of CD4+ CD25+ FoxP3+ Tregs frequency in the peripheral blood as well as lymph nodes of 4NQO-induced rat tongue carcinogenesis. The prevalence of Tregs was gradually increased in peripheral blood and regional lymph node according to disease progression during rat tongue carcinogenesis.

Regulatory T cells play vital roles in maintaining immune homeostasis, preventing autoimmune diseases and diminishing antitumor immunoresponse [17]. Increasing evidence demonstrated that the frequency and activity of CD4+ CD25+ FoxP3+ Tregs increased in the circulation and tumor microenvironment in patients with various types of cancer. The increase of Tregs was negatively correlated with tumor progression and prognosis [18, 19]. Recent data indicated that tumor immune response also played important role in oral carcinogenesis. Induction of immune surveillance is often observed in the early stage of OSCC [20]. However, the local and systemic cell-mediated response is suppressed following disease progression in cancer patients leading to the escape of tumor cells [21]. So far, the exact role of Tregs in the progression of OSCC remains unclear.

In this study, we used a good rat model to explore the role of Treg cells during oral carcinogenesis, which mimics the multistage carcinogenesis of human OSCC on morphological and biological concepts and allows the isolation of all stages under controlled conditions. We found that the frequency of Treg cells in peripheral blood was higher in squamous cell carcinoma rat than controls. Our results are in concordance with several previous studies in human cancer. CD4+ CD25+ Tregs from peripheral blood of OSCC patients had regulator phenotype, with higher detection of FoxP3 [14]. Next, we investigated the correlation of Tregs with tumor grades. Our data showed that Tregs in the peripheral blood occurred at an early stage of oral carcinogenesis and then the infiltration of Tregs gradually increased according to disease progression from moderate to severe oral dysplasia and squamous cell carcinoma. It suggested that the prevalence of circulating Tregs was significantly correlated with the tumor grades, and it may play an important role in oral cancer progression.

The tumor-draining lymph node is the critical initial site in which tumor antigens are typically first presented to the naive immune system and antitumor immune responses are initiate. It has outsize influence on host immune response [22]. A number of studies have identified CD4+ CD25+ Treg cells infiltration of draining lymph node in mouse models and human cancer. Munn et al. [23] proposed that the tumor-draining lymph node was a unique immunological environment where the presence of Tregs could mediate a suppressive effect on antitumor immune response. Moreover, accumulation of Tregs in draining lymph nodes was associated with poor prognosis in patients with colorectal cancer [24]. In addition, depletion of Tregs enhanced effector T cell responses in tumor-draining lymph nodes [25, 26]. These studies suggested that Treg cells in lymph nodes suppressed antitumor immune response. We questioned whether Treg cells infiltration in lymph nodes of oral cancer also suppress the host immune response. Our study showed that the percentage of CD4+ CD25+ Treg cells in lymph nodes was significantly higher from OSCC rat than in controls. Moreover, Tregs seemed to markedly accumulate more in rat lymph nodes than in peripheral blood. Next, we analyzed the relationship of Tregs frequency in lymph nodes with disease progression. The percentage of Tregs in lymph nodes was also observed to be increased by the increase in the tumor stage from moderate to severe oral dysplasia and squamous cell carcinoma. The novelty of this study was that Tregs was analyzed in lymph nodes in parallel with peripheral blood. Another research in gastric cancer also found significant elevated Tregs in PBMCs and tumor-draining lymph nodes [27]. These findings suggested that increased Tregs in lymph nodes might inhibit host immune response and favors malignant transformation and tumor progression.

In conclusion, the increase of Tregs in peripheral blood and tumor-draining lymph nodes of 4NQO-induced tongue carcinogenesis is correlated with tumor development. It might stand for an immunosuppressive mechanism in the pathogenesis of oral cancer. Further studies are needed to clarify the role and mechanism of Tregs regulation in the progression of oral cancer. The current study will provide new insights into the carcinogenesis of oral cancer.

## References

[1] Wolff KD, Follmann M, Nast A (2012). The diagnosis and treatment of oral cavity cancer. Dtsch Arztebl Int.

[2] Mithani SK, Mydlarz WK, Grumbine FL, Smith IM, Califano JA (2007). Molecular genetics of premalignant oral lesions. Oral Dis.

[3] Chang MC, Chiang CP, Lin CL, Lee JJ, Hahn LJ, Jeng JH (2005). Cell-mediated immunity and head and neck cancer: with special emphasis on betel quid chewing habit. Oral Oncol.

[4] Benoist C, Mathis D (2012). Treg cells, life history, and diversity. Cold Spring Harb Perspect Biol.

[5] Corsini E, Oukka M, Pieters R, Kerkvliet NI, Ponce R, Germolec DR (2011). Alterations in regulatory T-cells: rediscovered pathways in immunotoxicology. J Immunotoxicol.

[6] Chattopadhyay S, Chakraborty NG, Mukherji B (2005). Regulatory T cells and tumor immunity. Cancer Immunol Immunother.

[7] Perez SA, Karamouzis MV, Skarlos DV, Ardavanis A, Sotiriadou NN, Iliopoulou EG, Salagianni ML, Orphanos G, Baxevanis CN, Rigatos G, Papamichail M (2007). CD4+ CD25+ regulatory T-cell frequency in HER-2/neu (HER)-positive and HER-negative advanced-stage breast cancer patients. Clin Cancer Res.

[8] Mizukami Y, Kono K, Kawaguchi Y, Akaike H, Kamimura K, Suqai H, Kamimura K, Sugai H, Fujii H (2008). Localisation pattern of Foxp3+ regulatory T cells is associated with clinical behaviour in gastric cancer. Br J Cancer.

[9] Wang W, Hodkinson P, McLaren F, MacKinnon A, Wallace W, Howie S, Sethi T (2012). Small cell lung cancer tumour cells induce regulatory T lymphocytes, and patient survival correlates negatively with FOXP3+ cells in tumour infiltrate. Int J Cancer.

[10] Green VL, Irune E, Prasai A, Alhamarneh O, Greenman J, Stafford ND (2012). Serum IL10, IL12 and circulating CD4 + CD25high T regulatory cells in relation to long-term clinical outcome in head and neck squamous cell carcinoma patients. Int J Oncol.

[11] Leffers N, Gooden MJ, de Jong RA, Hoogeboom BN, ten Hoor KA, Hollema H, Boezen HM, van der Zee AG, Daemen T, Nijman HW (2009). Prognostic significance of tumor infiltrating T-lymphocytes in primary and metastatic lesions of advanced stage ovarian cancer. Cancer Immunol Immunother.

[12] Gao Q, Qiu SJ, Fan J, Zhou J, Wang XY, Xiao YS, Xu Y, Li YW, Tang ZY (2007). Intratumoral balance of regulatory and cytotoxic T cells is associated with prognosis of hepatocellular carcinoma after resection. J Clin Oncol.

[13] Yamagami W, Susumu N, Tanaka H, Hirasawa A, Banno K, Suzuki N, Tsuda H, Tsukazaki K, Aoki D (2011). Immunofluorescence-detected infiltration of CD4 + FOXP3+ regulatory T cells is relevant to the prognosis of patients with endometrial cancer. Int J Gynecol Cancer.

[14] Gasparoto TH, de Souza Malaspina TS, Benevides L, de Melo EJ, Jr CMR, Damante JH, Ikoma MR, Garlet GP, Cavassani KA, da Silva JS, Campanelli AP (2010). Patients with oral squamous cell carcinoma are characterized by increased frequency of suppressive regulatory T cells in the blood and tumor microenvironment. Cancer Immunol Immunother.

[15] Vitale-Cross L, Czerninski R, Amornphimoltham P, Patel V, Molinolo AA, Gutkind JS (2009). Chemical carcinogenesis models for evaluating molecular-targeted prevention and treatment of oral cancer. Cancer Prev Res (Phila).

[16] Kramer IR, Lucas RB, Pindborg JJ, Sobin LH (1978). Definition of leukoplakia and related lesions: an aid to studies on oral precancer. Oral Surg Oral Med Oral Pathol.

[17] Whiteside TL (2012). What are regulatory T cells (Treg) regulating in cancer and why?. Semin Cancer Biol.

[18] Betts G, Jones E, Junaid S, El-Shanawany T, Scurr M, Mizen P, Kumar M, Jones S, Rees B, Williams G, Gallimore A, Godkin A (2012). Suppression of tumour-specific CD4 + T cells by regulatory T cells is associated with progression of human colorectal cancer. Gut.

[19] Chen KJ, Lin SZ, Zhou L, Xie HY, Zhou WH, Taki-Eldin A, Zheng SS (2011). Selective recruitment of regulatory T cell through CCR6-CCL20 in hepatocellular carcinoma fosters tumor progression and predicts poor prognosis. PLoS One.

[20] Schwarz S, Butz M, Morsczeck C, Reichert TE, Driemel O (2008). Increased number of CD25 FoxP3 regulatory T cells in oral squamous cell carcinomas detected by chromogenic immunohistochemical double staining. J Oral Pathol Med.

[21] Gaur P, Qadir GA, Upadhyay S, Singh AK, Shukla NK, Das SN (2012). Skewed immunological balance between Th17 (CD4(+)IL17A (+)) and Treg (CD4 (+)CD25 (+)FOXP3 (+)) cells in human oral squamous cell carcinoma. Cell Oncol (Dordr).

[22] Shu S, Cochran AJ, Huang RR, Morton DL, Maecker HT (2006). Immune responses in the draining lymph nodes against cancer: implications for immunotherapy. Cancer Metastasis Rev.

[23] Munn DH, Mellor AL (2006). The tumor-draining lymph node as an immune privileged site. Immunol Rev.

[24] Deng L, Zhang H, Luan Y, Zhang J, Xing Q, Dong S, Wu X, Liu M, Wang S (2010). Accumulation of foxp3+ T regulatory cells in draining lymph nodes correlates with disease progression and immune suppression in colorectal cancer patients. Clin Cancer Res.

[25] Coe D, Begom S, Addey C, White M, Dyson J, Chai JG (2010). Depletion of regulatory T cells by anti-GITR mAb as a novel mechanism for cancer immunotherapy. Cancer Immunol Immunother.

[26] Baba J, Watanabe S, Saida Y, Tanaka T, Miyabayashi T, Koshio J, Ichikawa K, Nozaki K, Koya T, Deguchi K, Tan C, Miura S, Tanaka H, Tanaka J, Kagamu H, Yoshizawa H, Nakata K, Narita I (2012). Depletion of radio-resistant regulatory T cells enhances antitumor immunity during recovery from lymphopenia. Blood.

[27] Mizukami Y, Kono K, Kawaguchi Y, Akaike H, Kamimura K, Sugai H, Fujii H (2008). CCL17 and CCL22 chemokines within tumor microenvironment are related to accumulation of Foxp3+ regulatory T cells in gastric cancer. Int J Cancer.

